# Pneumococcal carriage in households in Karonga District, Malawi, before and after introduction of 13-valent pneumococcal conjugate vaccination

**DOI:** 10.1016/j.vaccine.2018.10.021

**Published:** 2018-11-19

**Authors:** Ellen Heinsbroek, Terence Tafatatha, Amos Phiri, Todd D Swarthout, Maaike Alaerts, Amelia C Crampin, Christina Chisambo, Oddie Mwiba, Jonathan M Read, Neil French

**Affiliations:** a Institute of Infection and Global Health, University of Liverpool, Liverpool, UK; b Karonga Prevention Study, Chilumba, Malawi; c Malawi-Liverpool-Wellcome Trust Clinical Research Programme, College of Medicine, University of Malawi, Blantyre, Malawi; d Clinical Sciences Department, Liverpool School of Tropical Medicine, Liverpool, UK; e London School of Hygiene and Tropical Medicine, London, UK; f Centre for Health Informatics Computing and Statistics, Lancaster Medical School, Lancaster University, Lancaster, UK

**Keywords:** *Streptococcus pneumoniae*, Carriage, Infant, Africa, Cohort studies, Pneumococcal conjugate vaccine

## Abstract

**Background:**

Thirteen-valent pneumococcal conjugate vaccine (PCV13) was introduced in Malawi in November 2011 and is offered to infants at 6, 10 and 14 weeks of age as part of routine immunisation. PCV13 is expected to reduce vaccine type (VT) nasopharyngeal carriage, leading to reduced transmission and herd protection.

**Methods:**

We compared pneumococcal carriage in rural Karonga District, Malawi, pre-vaccine in 2009–2011 and post-vaccine in 2014 using a combination of cross-sectional and longitudinal analyses. Nasopharyngeal swabs were collected from a cohort of mother-infant pairs and household members <16 years. Pneumococci from 2009 to 2011 were serogrouped using latex agglutination and serotyped by Quellung reaction. In 2014, latex agglutination was used for both steps. Carriage prevalence ratios using prevalence data from before and after vaccine introduction were calculated by log-binomial regression, adjusted for age, seasonality and household composition. Participating infants in 2014 received PCV13 as part of routine immunisation.

**Results:**

VT carriage prior to PCV-13 introduction was 11.4%, 45.1%, 28.2%, 21.2% and 6.6% for 6-week old infants, 18-week old infants, children 1–4 years, children 5–15 years and mothers, respectively. After vaccine introduction, VT carriage decreased among vaccinated 18-week old infants (adjusted prevalence ratio 0.24 (95%CI 0.08–0.75)), vaccinated children 1–4 years (0.54 (0.33–0.88)), unvaccinated children 5–15 years (0.37 (0.17–0.78)) and mothers (0.34 (0.15–0.79)). No decrease in VT carriage was observed for 6-week old infants too young to be vaccinated (1.07 (0.38–3.02)) and PCV-13 ineligible children 1–4 years (0.84 (0.53–1.33)). Non-VT carriage increased only among vaccinated children 1–4 years (1.58 (1.21–2.06)).

**Conclusions:**

There is evidence of reduced VT pneumococcal carriage three years after vaccine introduction in this rural Malawian population with good vaccine coverage using a 3 + 0 schedule. However carriage was sustained among 6-week-old infants and PCV13 ineligible 1–4 year olds, and there was some indication of serotype replacement in vaccinated 1–4 year olds.

## Highlights

• Pneumococcal carriage of any serotype remained high three years post vaccine introduction.
• Early acquisition of pneumococcal carriage in infants continued post vaccine introduction.
• Vaccine-type carriage was lower in vaccinated and unvaccinated individuals.
• Non-vaccine-type carriage was higher post vaccine introduction in vaccinated children.
• There is evidence of herd protection and serotype replacement in this population.

## 1 Introduction

*Streptococcus pneumoniae* (pneumococcus) is a leading cause of childhood morbidity and mortality worldwide. The nasopharynx is the primary portal for entry and source for transmission of the pneumococcus. Although mostly asymptomatic, nasopharyngeal carriage is thought to be a pre-requisite for disease [1]. Asymptomatic carriers are also the main source of pneumococcal transmission, with person-to-person spread occurring in close contact [1]. Pneumococcal acquisition occurs very early in life in developing countries, with an observed median time to first acquisition of only 38.5 days, 45.5 days, 59 days or 8 weeks after birth reported in Kenya, Thailand-Myanmar, Malawi and Bangladesh respectively [2], [3], [4], [5]. This early and intense exposure in infancy is likely to play a role in the high disease incidence experienced in these settings.

Pneumococcal conjugate vaccines (PCV) have been shown effective against vaccine type (VT) invasive pneumococcal disease (IPD) [6], [7], [8]. PCV reduces nasopharyngeal carriage in vaccinated individuals [9], allowing for a herd effect, as observed in many high-income countries [10], [11], [12] and some low-income countries [8], [13]. Serotype replacement is a concern [14], [15], but although increases in non-vaccine type (NVT) IPD have been observed after PCV introduction in most study sites, rates of overall IPD have dropped as a result of a decrease in VT pneumococci [16]. Evidence is mainly available for high-income countries, however introduction of PCV in low-income countries could result in a higher rate of serotype replacement due to a high background rate of pneumococcal carriage. Also, the HIV-burden in many countries could have an effect on serotype replacement, as carriage of NVT pneumococci was found to be high in HIV-infected adults prior to PCV13 introduction despite established antiretroviral treatment [17].

Thirteen-valent pneumococcal conjugate vaccine (PCV13) was introduced in the Malawian infant immunisation programme in November 2011 using a “3 + 0” schedule with doses given at 6, 10 and 14 weeks. Initial PCV13 catch-up vaccination was conducted at the time of introduction with infants <1 year of age at date of first dose receiving 3 doses at 1-month intervals. Vaccine coverage with 3 doses PCV13 at 1 year of age among those eligible for PCV13 in the birth cohort was 89.4% in Karonga District, Malawi, in 2014 [18]. Vaccine coverage with 3 doses PCV13 in the catch-up cohort was 49.8% [18]. We compared pneumococcal carriage in Karonga District, Malawi, before and after introduction of PCV13, in order to review the effect of the pneumococcal vaccination programme on VT and NVT carriage.

## 2 Materials and methods

### 2.1 Study population and design

The study was conducted in the area covered by the Karonga Health and Demographic Surveillance System (HDSS) in northern Malawi [19]. Established in 2002, the HDSS covers an area of 135 km^2^ and provides continuous population surveillance with all births, deaths and migrations recorded. The population size was 34,111 in January 2009, increasing approximately 2.5% per annum. The area has an annual birth cohort of about 1500. In 2010 life expectancy at birth was 69.4 years, with infant and under-five mortality rates of 35.0 and 59.1 per 1000 live births, respectively [19]. Main sources of income in the KHDSS area are subsistence farming, fishing and small-scale trading. HIV-prevalence in women of childbearing age ranged between 3% in women 15–24 years and 16% in women 30–39 years in 2008/2009 [20], remaining relatively stable between 2008 and 2014 [21]. ART has been available in the government clinic within the study catchment area since 2006; ART uptake in the HDSS was estimated to be at least 60% of those eligible in mid-2008 [20].

Longitudinal household pneumococcal carriage studies were conducted in 2009–2011 before PCV13 introduction and 2.5 years post PCV13 introduction in 2014. Here we present a combination of cross-sectional and longitudinal data analyses to compare pneumococcal carriage prevalence and pneumococcal acquisition in infants between the pre- and post-PCV13 periods. An overview of the data collection and analyses can be found in Supplementary Fig. 1. A longitudinal design was chosen to study pneumococcal transmission within households, results for which have been published elsewhere [5].

The methods and results of the 2009–2011 study have previously been published [5]. Briefly, pregnant women living in the HDSS area were recruited from antenatal clinics in two rural hospitals between January 2009 and December 2010. These women were then followed up at their home after delivery, collecting nasopharyngeal swabs from the mother, the infant, and other household members willing to participate at 6, 10, 14, 18, 22, 26, 30, 34, 40, 46, and 52 weeks of the infant’s age.

In 2014, recruitment of mother-infant pairs took place in the postnatal clinics of two rural hospitals between April and July of the same year. All mother-infant pairs living within the HDSS area who were discharged within one week of delivery were eligible for inclusion in the study. The first 44 mother-infant pairs recruited were sampled longitudinally: nasopharyngeal swabs were collected at their household from the infant, mother, and any other children 1–4 years in the household at 6, 8, 10, 12, 14, 16, 18 weeks of the infant’s age. The remaining mother-infant pairs recruited were sampled only once at 6 weeks of the infant’s age. This was done as a cross-sectional component to minimise logistical challenges associated with longitudinal sample collection while increasing the number of samples and statistical power of the study in calculating carriage at 6 weeks of age (prior to receiving first dose of PCV13).

Older children (5–15 years) willing to participate were included only in the cross-sectional component of the 2014 study. No adults other than the mother were included in the 2014 study. This decision was based on experience from the 2009–2011 study demonstrating frequent refusal of adults other than the mother and poor retention of older children in longitudinal sampling. HIV-status of the mother was transcribed from her “health passport” (patient-held health record) or the mother was asked for her HIV-status verbally if the health passport was unavailable. Only the results for HIV-negative mothers were included in the analyses: previous work showed that pneumococcal carriage was higher in HIV-infected than in HIV-uninfected mothers [5]. HIV exposure was not found in previous work to be associated with differences in pneumococcal carriage prevalence or serotype distribution in childhood [5]; therefore all samples from children were included regardless of the HIV status of the mother.

Recruited infants were offered routine vaccinations, including PCV13 in 2014, at 6, 10, and 14 weeks of age. Vaccination status of other children 1–4 years in the household was obtained from HDSS records. Follow-up ceased if the infant died or moved outside the study area, consent was withdrawn or there was a failure to sample on two sequential visits.

### 2.2 Laboratory procedures

Nasopharyngeal samples were collected, processed and analysed per WHO recommendations [22] as described previously [5]. A calcium alginate swab (Medical Wire & Equipment, Corsham, UK) was inserted into the posterior nasopharynx. The swab was transported in skim milk-tryptone-glucose-glycerol medium. Inoculated vials were stored at −20 °C within 6 h of collection, and were frozen at −80 °C within days until tested. Samples (30 µL) were cultured on gentamicin (5 µg/mL) sheep blood agar plates and incubated overnight at 37 °C with 5% carbon dioxide. Pneumococci were identified by morphology and sensitivity for optochin. A single colony was selected from the primary plate and a secondary plate was grown to obtain pure growth. Pneumococci from 2009 to 2011 were serogrouped using latex agglutination and serotyped by Quellung reaction. In 2014, latex agglutination was used for both steps. Both assays used standard antisera (Statens Serum Institute Denmark). The 13-valent latex serotyping kit was used to identify individual VT (1, 3, 4, 5, 6A, 6B, 7F, 9 V, 14, 18C, 19A, 19F, 23F) and provide some detail on NVT serogroup (e.g. 7A/7B/7C). Serotyping for NVT was not done due to budget constraints.

### 2.3 Cross-sectional analysis comparing the pre-and post-PCV13 period

Analyses comparing the pre- and post-PCV13 period were season-matched: only the pre-PCV13 samples collected in April-August of each year were included because post-PCV13 sampling was limited to these months. Seasonality was shown in the pre-PCV13 study to be a strong predictor of pneumococcal carriage, with carriage being highest in the cold season (May-August) [5]. Carriage prevalence in the pre- and post-PCV13 periods was calculated for infants at 6 weeks (prior to receiving PCV13 in 2014), infants at 18 weeks (post receiving PCV13 in 2014), children 1–4 years, children 5–15 years and HIV-negative mothers. To increase the power of the analysis, repeated longitudinal samples for the mothers and children 1–15 years in the household were used in the cross-sectional analysis.

Carriage prevalence ratios (PR) were calculated for the periods before and after vaccine introduction. Potential confounders were identified by testing the association between variables and the vaccine period and included in the multivariate models when p < 0.1. Adjusted prevalence ratios (APR) were calculated using log-binomial regression, or Poisson regression with robust standard errors if the log-binomial regression failed to converge [23]. Mixed models with individual-level random effects were fitted to examine within-person clustering as a result of the longitudinal sampling of mothers and children 1–15 years.

### 2.4 Longitudinal analyses

Pneumococcal acquisition in infants was assessed by survival analysis including Kaplan-Meier plots, log-rank tests, and cox proportional hazard models adjusted for the number of children <5 years in the household [24]. An episode of carriage was defined as isolation of a pneumococcus from one or more consecutive samples. New acquisition was defined as the identification of a serotype not identified at the previous two sampling times. NVT serogroups without further distinction (e.g. 7A/7B/7C) were regarded to be the same serotype. Pneumococcal isolates identified in the first sample were regarded as new acquisitions. The date of acquisition was defined as the midpoint between the last negative and the first positive result. To allow for comparison of mean time to first acquisition between 2009 and 2011 and 2014, only samples from weeks 6, 10, 14 and 18 were used in the survival analyses (weeks sampled in both 2009–2011 and 2014). The reduction in pneumococcal acquisition at 10, 14 and 18 weeks was calculated as 1-RR.

### 2.5 Ethics

Informed written consent was obtained from participating mothers. Ethical approval was granted by the National Health Sciences Research Committee in Malawi (#490, #1232), the London School of Hygiene and Tropical Medicine (# 5345) and the University of Liverpool (#670) ethics committees.

## 3 Results

### 3.1 Study participants and samples

**Fig. 1:**
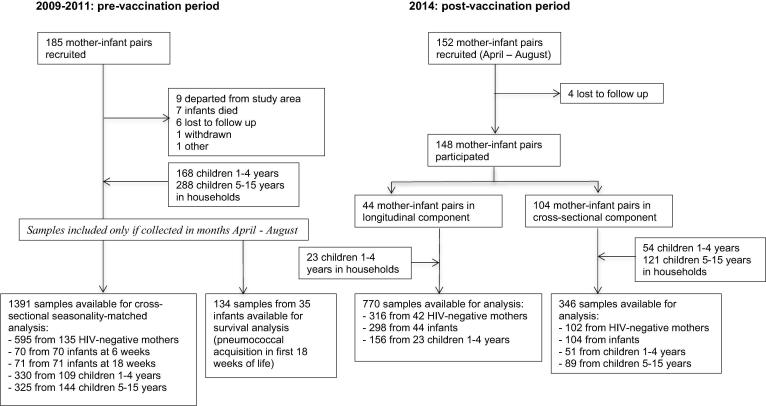
Flowchart of recruitment and availability of nasopharyngeal samples of mothers, infants, and children 1–15 years in Karonga District, Malawi in the pre- and post-PCV13 periods.

Fig. 1 provides a flowchart of recruitment and availability of samples in the pre- and post-PCV13 period. In 2009–11, 185 mother infant pairs were recruited. Follow-up ceased prematurely for 24 infants (13.0%) because they departed from the study area (n = 9), died within the first year of life (n = 7), were lost to follow-up (n = 6), were withdrawn from the study (n = 1) or left for other reasons (n = 1). For this seasonality-matched cross-sectional analysis comparing pneumococcal prevalence in the pre- and post-PCV13 period, 1391 samples were available from 70 6-week old infants, 71 18-week old infants, 109 children 1–4 years, 144 children 5–15 years and 135 HIV-negative mothers recruited from a total of 166 households. In total 134 samples from 35 infants were available for the survival analysis comparing pneumococcal acquisition in the first 18 weeks of life in the pre- and post-PCV13 period.

In 2014, 152 mother-infant pairs were recruited; four mother-infant pairs were lost to follow up and 148 mother-infant pairs participated in the study: the first 44 in the longitudinal component, the latter 104 in the cross-sectional component. Documented HIV test results were available for 88.5% (131/148) mothers. Four (2.7%) mothers were HIV-positive (two with documented, two with verbal report). Of the HIV-negative mothers, 97.9% (141/144) were tested within the last year. In the longitudinal component there were 23 children 1–4 years in participating households; at least one nasopharyngeal sample was collected from all. In the cross-sectional component there were 54 children 1–4 years and 121 children 5–15 years in the participating households: a nasopharyngeal sample was collected from 51 (94.4%) and 89 (73.6%) respectively. Four children were included as infant in the pre-PCV13 and as a child 1–4 years in the post-PCV13 period. Six samples were lost, leaving 1116 samples for analysis; 770 collected in the longitudinal component, 346 in the cross-sectional component of the study.

All infants participating in the 2014 study received PCV13 at 6, 10, 14 weeks of age. Among the 77 children 1–4 years, 25 (32.5%) were age-eligible for vaccination in the birth cohort and had received full vaccination with PCV13. Twenty-eight children (36.4%) were age-eligible for vaccination in the catch-up cohort; 15 (53.6%) had received full vaccination, 4 (14.3%) partial vaccination and 9 (32.1%) no vaccination. Twenty-three children (29.9%) were not age-eligible for PCV13. One child’s vaccine eligibility was unknown. No children ≥5 years or adults received PCV13.

### 3.2 Characteristics of participants

Table 1 shows the characteristics of participants in the pre- and post-PCV13 periods. Participating children 5–15 years were older, HIV-negative mothers were younger and there were fewer households with children <5 years other than the recruited infant in the post-PCV13 period. Pneumococcal prevalence ratios were adjusted for age and number of children <5 years in the household, factors known to be associated with pneumococcal carriage.

**Table 1:** Characteristics of participating mothers, infants, and children 1–15 years in Karonga District, Malawi in the pre- and post-PCV13 periods.

	Pre-PCV13 period (2009–11)	Post-PCV13 period (2014)	p-value^1^
Female sex			
Infants 6 weeks	35/70 (50.0%)	75/146 (51.4%)	0.97
Infants 18 weeks	33/71 (46.5%)	26/44 (59.1%)	0.26
Children 1–4 years	66/109 (60.6%)	12/29 (41.4%) (unvaccinated)	0.10
		15/38 (39.5%) (vaccinated)	0.04
		27/74 (36.5%) (all)	0.002
Children 5–15 years	72/144 (50.0%)	40/89 (44.9%)	0.54
HIV-negative mothers	135/135 (100%)	144/144 (100%)	–

Age in years (mean, sd)			
Children 1–4 years	2.7, sd = 0.9	3.5, sd = 0.6 (unvaccinated)	<0.001
		2.1, sd = 0.7 (vaccinated)	<0.001
		2.7, sd = 1.0 (all)	0.99
Children 5–15 years	7.8, sd = 2.6	8.5, sd = 3.0	0.002
HIV-negative mothers	26.3, sd = 6.9	24.2, sd = 6.3	<0.001

Number of children <5 years other than the recruited infant (households)	0: 45/166 (27.1%)	0: 79/148 (53.4%)	
	1: 92/166 (55.4%)	1: 61/148 (41.2%)	<0.001
	2: 29/166 (17.5%)	2: 8/148(5.4%)	

Information included for individuals with at least one sample result.

sd: standard deviation

1 p-values for the comparison between the pre-PCV13 and post-PCV13 period; using the Pearson’s χ**^2^** test for categorical data and the Student’s *t*-test for numerical data.

### 3.3 Pneumococcal carriage prevalence in the pre- and post-PCV13 period

**Fig. 2:**
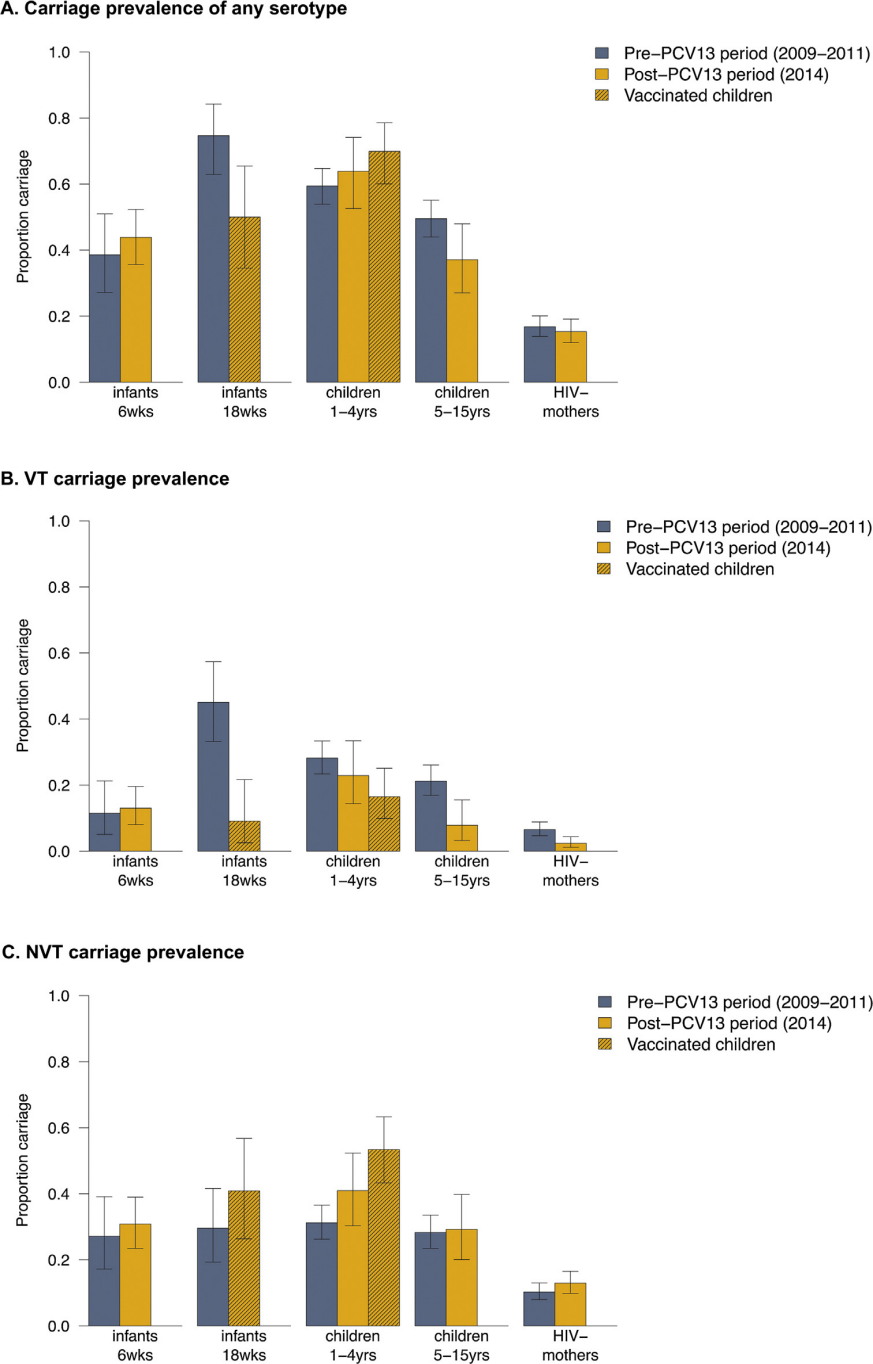
Prevalence of carriage of any serotype (A), VT carriage (B) or NVT carriage (C) among mothers, infants and children 1–15 years in Karonga District, Malawi, in the pre- and post-PCV13 periods.

**Fig. 3:**
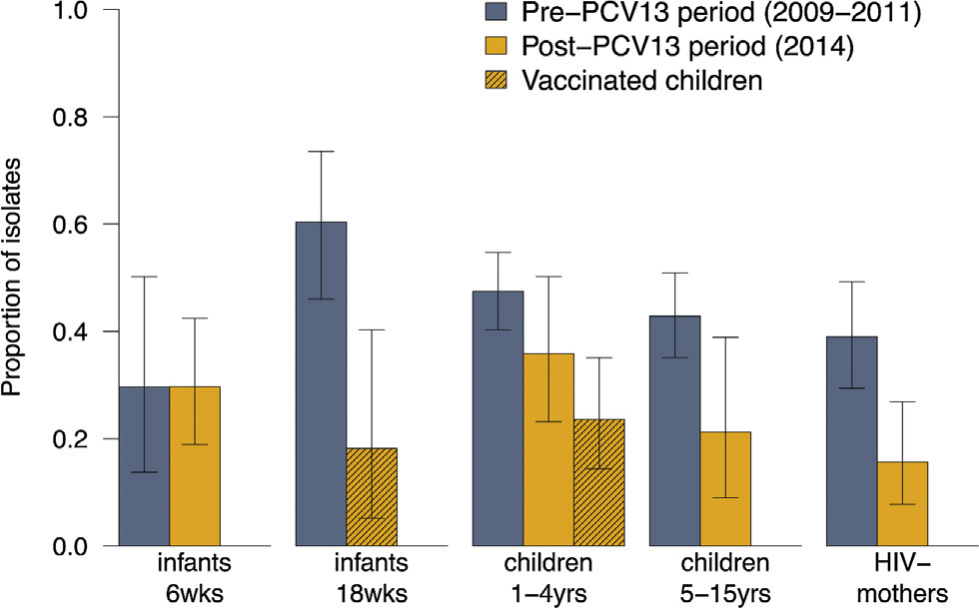
Proportion of VT isolates among pneumococcal carriers among mothers, infants and children 1–15 years in Karonga District, Malawi, in the pre- and post-PCV13 periods.

Fig. 2 shows the pneumococcal carriage prevalence by age group in the pre- and post-PCV13 periods. Fig. 3 shows the proportion of VT isolates among pneumococcal carriers. The proportion of VT isolates among pneumococcal carriage was lower in 2014 than in 2009–2011 for all age groups, except 6-week old infants (Fig. 3).

Table 2 shows the crude and adjusted prevalence ratios for the pre- and post-PCV13 period. After vaccine introduction, VT carriage decreased among vaccinated 18-week old infants and vaccinated children 1–4 years. Although decreased, VT carriage remained common in vaccinated 1–4 years at 16.5%. VT carriage also decreased in children 5–15 years and mothers. A decrease in VT carriage from 28.2% to 22.9% was observed in unvaccinated children 1–4 years, but this was not found to be significant. No decrease was observed for 6-week infants. NVT carriage increased among vaccinated children 1–4 years only; from 31.2% to 53.4%.

**Table 2:** Carriage prevalence and prevalence ratio for pneumococcal carriage among mothers, infants and children 1–15 years in Karonga District, Malawi, in the pre- and post-PCV13 periods.

	Vaccine status, 2014^1^	Carriage prevalence, 2009–2011 (pre PCV13 introduction)	Carriage prevalence, 2014 (post PCV13 introduction)	Crude prevalence ratio (95%CI)	Adjusted prevalence ratio (95%CI)^3^
*All serotypes*					
Infants, 6 wks	unvaccinated	27/70 (38.6%)	64/146 (43.8%)	1.14 (0.80–1.61)	0.98 (0.66–1.47)
Infants, 18 wks	fully vaccinated	53/71 (74.7%)	22/44 (50.0%)	0.67 (0.48–0.93)	0.60 (0.42–0.86)^4^
Children 1–4 yrs^2^	unvaccinated	196/330 (59.4%)	53/83 (63.9%)	1.08 (0.89–1.29)	1.06 (0.86–1.30)^4^
	fully vaccinated	–	72/103 (70.0%)	1.18 (1.01–1.37)	1.08 (0.91–1.28)^4^
	all	–	141/207 (68.1%)	1.15 (1.01–1.30)	1.10 (0.97–1.25)^4^
Children 5–15 yrs	unvaccinated	161/325 (49.5%)	33/89 (37.1%)	0.75 (0.56–1.00)	0.73 (0.55–0.97)^4^
HIV-negative mothers	unvaccinated	100/595 (16.8%)	64/418 (15.3%)	0.92 (0.69–1.22)	0.75 (0.47–1.17)

*VT*					
Infants, 6 wks	unvaccinated	8/70 (11.4%)	19/146 (13.0%)	1.14 (0.52–2.47)	1.07 (0.38–3.02)
Infants, 18 wks	fully vaccinated	32/71 (45.1%)	4/44 (9.1%)	0.20 (0.08–0.53)	0.24 (0.08–0.75)^5^
Children 1–4 yrs^2^	unvaccinated	93/330 (28.2%)	19/83 (22.9%)	0.81 (0.53–1.25)	0.84 (0.53–1.33)^5^
	fully vaccinated	–	17/103 (16.5%)	0.59 (0.37–0.93)	0.54 (0.33–0.88)^4^
	all	–	37/207 (17.9%)	0.63 (0.45–0.89)	0.63 (0.45–0.90)^4^
Children 5–15 yrs	unvaccinated	69/325 (21.2%)	7/89 (7.9%)	0.37 (0.18–0.78)	0.37 (0.17–0.78)
HIV-negative mothers	unvaccinated	39/595 (6.6%)	10/418 (2.4%)	0.37 (0.19–0.73)	0.34 (0.15–0.79)

*NVT*					
Infants, 6 wks	unvaccinated	19/70 (27.1%)	45/146 (30.8%)	1.14 (0.72–1.79)	0.95 (0.57–1.56)
Infants, 18 wks	fully vaccinated	21/71 (29.6%)	18/44 (40.9%)	1.38 (0.83–2.29)	0.91 (0.47–1.77)
Children 1–4 yrs^2^	unvaccinated	103/330 (31.2%)	34/83 (41.0%)	1.31 (0.97–1.78)	1.24 (0.88–1.74)^4^
	fully vaccinated	–	55/103 (53.4%)	1.71 (1.34–2.18)	1.58 (1.21–2.06)^4^
	all	–	104/207 (50.2%)	1.61 (1.30–1.99)	1.50 (1.22–1.86)
Children 5–15 yrs	unvaccinated	92/325 (28.3%)	26/89 (29.2%)	1.03 (0.72–1.49)	1.03 (0.71–1.49)
HIV-negative mothers	unvaccinated	61/595 (10.3%)	54/418 (12.9%)	1.27 (0.90–1.79)	0.99 (0.57–1.73)

1 Fully vaccinated: 3 doses of PCV13; unvaccinated: no doses of PCV13 received; all: including partially vaccinated children and children with unknown vaccination status, in addition to the fully vaccinated and unvaccinated.

2 Children 1–4 years: carriage in 2014 of unvaccinated, fully vaccinated and all children 1–4 years is compared to carriage in 2009–2011 (pre-PCV13 period; all unvaccinated). Unvaccinated children 1–4 years in 2014: this includes samples from 9 children who were eligible for PCV13 in the catch-up cohort but received no vaccination, and 23 children who were not age-eligible for PCV13.

3 Adjusted for month of sample collection, number of children 1–4 years in the household, age, and within-person clustering (mother only). Using a generalized linear mixed model, there was negligible individual-level variance for children 1–4 years and 5–15 years of age (σ2 < 0.01), hence results from a (non-mixed) generalized linear model were reported. Using a generalized linear mixed model, the individual-level variance for mothers was 0.68 (all serotypes)/1.25 (PCV13)/1.03 (non-PCV13).

4 Log-binomial regression model data did not to converge so results of a Poisson model with robust standard errors are presented.

In the post-PCV13 period, VT isolated were 19F (14 individuals), 6B (13) and 6A (10), 19A (7), 9 V (5), 14 (5), 3 (4), 18C (2) and 5(1).

### 3.4 Pneumococcal acquisition in infants

**Fig. 4:**
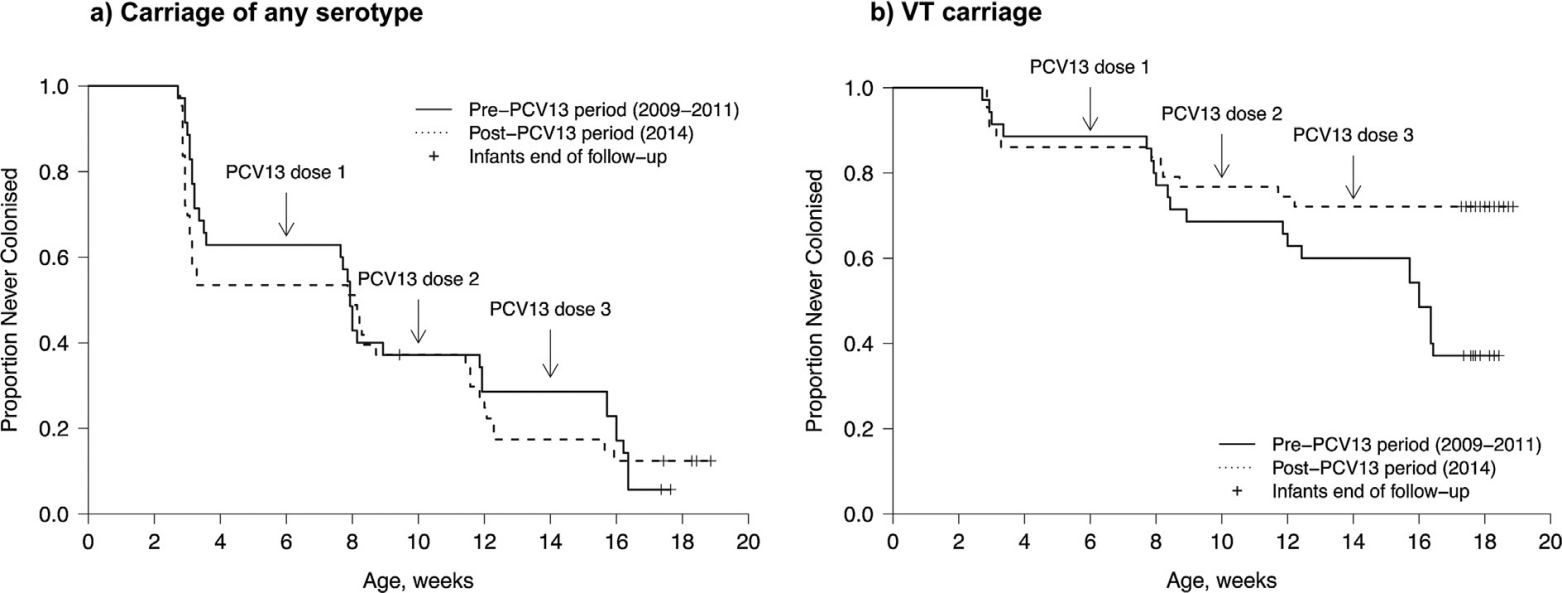
Kaplan-Meier plot for time to first pneumococcal carriage acquisition of any serotype (A) or a vaccine serotype (B) in infants in Karonga District, Malawi, pre and post introduction of PCV13. Observations in the pre-PCV13 period right-truncated at 18 weeks (maximum observation period post-PCV13 period).

Fig. 4 shows the time to first pneumococcal carriage acquisition in infants in the pre- and post-PCV13 periods. For acquisition of any serotype, no difference was observed between the pre- and post-PCV13 period (median time 55.5 vs. 56.5 days, p = 0.71). After adjustment for the number of children 1–4 years in the household, the hazard ratio for pneumococcal colonization in the post-PCV13 compared to the pre-PCV13 period was 1.10 (95%CI 0.63–1.91). For VT carriage, a difference was observed between the pre- and post-PCV13 period, with 62.9% (22/35) vs. 27.9% (12/43) of infants acquiring a VT pneumococcus at least once within the first 18 weeks of life (p = 0.008). After adjustment for the number of children 1–4 years in the household, the hazard ratio for VT pneumococcal colonization in the post-PCV13 compared to the pre-PCV13 period was 0.37 (95%CI 0.17–0.81), indicating a 63% reduction.

Observations in the pre-PCV13 period right-truncated at 18 weeks (maximum observation period post-PCV13 period).

Incidence of infant VT carriage acquisition in weeks 6–10 was 24.2% (8/33) in the pre-PCV13 period and 9.3% (4/43) in the post-PCV13 period, implying a 62% reduction in VT carriage acquisition for one dose of PCV13. Incidence in weeks 10–14 was 20.6% (7/34) in the pre-PCV13 period and 4.9% (2/41) in the post-PCV13 period, implying a 76% reduction in VT carriage acquisition for two doses of PCV13. Incidence in weeks 14–18 was 40.6% (13/32) in the pre-PCV13 period and 2.3% (1/44) in the post-PCV13 period, implying a 94% reduction in VT carriage acquisition for three doses of PCV13. Combining the results for all weeks, the VT carriage reduction for at least one dose of PCV13 was 81%.

In 2014, infants acquired pneumococci of any serotype at an earlier age when living with children 1–4 years in the household (Supplementary Fig. 2). No evidence was found for a difference in first VT acquisition between infants living with vaccinated or unvaccinated children 1–4 years (p = 0.73, results not shown).

## 4 Discussion

This is the first published study on pneumococcal carriage prevalence after routine introduction of PCV13 in a vaccine-naive sub-Saharan country using a 3 + 0 schedule. Previous studies were conducted in Kenya [25], [26], The Gambia [27] and South Africa [28], but different vaccines and/or schedules were used (Supplementary Table 1). We provide evidence for a reduction in VT carriage three years after PCV13 introduction in this rural Malawi population. A direct vaccine effect was obtained immediately after vaccination: occurrence of any infant pneumococcal acquisition in the first 18 weeks was reduced by 63% when comparing the pre- and post-PCV13 period. Whilst there is evidence for herd protection in older children and mothers, VT carriage remained high among 6-week old infants too young to be vaccinated and unvaccinated children 1–4 years. NVT carriage increased among vaccinated children 1–4 years, providing evidence for moderate levels of serotype replacement in this group.

Although a reduction of VT carriage was observed in vaccinated children 1–4 years, carriage prevalence remained a relatively high 17%. Similar results were found in studies in Kenya [25], The Gambia [27] and South Africa [28], also conducted two years post PCV introduction; although VT carriage was reduced post vaccine introduction in all three sites, VT carriage was sustained at 13% in children <5 years in Kenya, at 24% in <2 year-olds and 29% in 2–5 year-olds in South Africa, and at 18% in 6–11 month-olds in the Gambia (Supplementary Table 1). These estimates are much higher than the <1–4% VT carriage in vaccinated children observed after PCV introduction in high-income countries [29], [30], [31]. Results from a more recent study conducted in Kenya show that VT carriage in children <5 years further reduced to 9% in the five years post PCV introduction, suggesting an ongoing but delayed effect [26]. Results from a recent cross-sectional survey in Blantyre, an urban city in southern Malawi, also show that VT carriage can be sustained years after PCV13 introduction: VT carriage prevalence of 20% was observed in vaccinated 3–6 year-olds in 2017, more than five years post introduction of the vaccine [32].

We have evidence of an indirect effect in older children and adults, but indirect effects may be absent or perhaps more plausibly delayed in unvaccinated younger age groups. VT carriage was reduced in unvaccinated children 1–4 years, but this reduction did not reach statistical significance, and no reduction was observed in the pre-vaccine 6-week age group. Differences in herd benefit are likely a result of different contact patterns between age groups [33], in combination with differences in naturally acquired immunity. Exposure analyses from the pre-PCV13 period suggest that infant-to-mother and infant-to-sibling transmission frequently occurs [5] and it is likely that this will have been an important contributing factor to the herd effect in older children and mothers observed. Children <5 years do not yet have optimal natural acquired immunity, yet do experience a high force of infection [32] which could explain the delay of herd benefit observed in this young age group. Results from the carriage study in Blantyre, southern Malawi suggest that development of indirect protection in unvaccinated children is delayed, but does develop over time: VT carriage among unvaccinated children 5–10 years was 28% in 2015, but dropped to 10% in 2017 [32]. Our study was conducted only 2.5 years after PCV13 introduction; it is probable that a further drop in VT carriage in all age groups has occurred now that the vaccinated population has increased in size.

Sustained VT carriage in vaccinated children is likely to be a result of re-colonisation after waning mucosal immunity [32], and suggests that a booster dose is essential to prolong anti-carriage immunity. A ‘2 + 1′ schedule with the booster dose of PCV13 given at 9 months to coincide with measles vaccination should be assessed for its effect on delaying the waning of mucosal immunity in vaccinated individuals, and for its subsequent effect on herd immunity, particularly in the unvaccinated younger age groups. In addition, a further booster dose may be required in the second year of life or at pre-school age to sustain anti-carriage immunity. Although this would increase vaccine costs this may be essential to generate herd protection and may only be required until herd protection is achieved.

Countries newly introducing PCV should further consider implementing a catch-up campaign with a broader age range than was implemented in Malawi. In Malawi, a 3-dose catch-up campaign was conducted for infants up to one year of age, for which moderate coverage was achieved (49.8% for 3 doses PCV13) in Karonga District [18]. In Kilifi County in Kenya, where PCV10 was introduced in 2011 with a catch-up campaign for children <5 years, VT carriage declined in all age groups within 6 months of PCV10 introduction [25], suggesting that a broader catch-up campaign is essential to obtain a more immediate herd impact.

Any before/after study suffers from potential bias from underlying temporal change in carriage prevalence independent of the vaccine effect, which may be difficult to assess. There were no other known interventions, secular changes or social patterning over this period that would have impacted on pneumococcal carriage. Although a cookstove trial was conducted in this population in 2014 [34], there was no evidence that the intervention clusters had different rates of carriage (unpublished results). We are confident that a vaccine effect largely explains our measured differences. VT carriage was reduced most in the vaccinated groups and there was some evidence of dose response with greater reductions observed with more doses of vaccine. We used slightly different recruitment methods in 2014 based on experience in 2009–11, but compared seasonally-matched periods.

Our laboratory procedures did not allow for detection of simultaneous colonization with multiple serotypes and previous work suggests this is common in this population [35], [36]. This will not have influenced our comparisons between the pre- and post-PCV13 period, but will have decreased the accuracy of our estimates of carriage prevalence. Our study was not powered to assess changes in individual serotypes and our laboratory procedures did not differentiate individual NVT; we can therefore not assess whether individual serotypes have increased or decreased since PCV13 introduction.

In conclusion, three years post PCV13 introduction in Malawi, we observed changes in pneumococcal epidemiology consistent with direct and herd effects. Despite the observed direct and herd effects, VT carriage was sustained at 17% in vaccinated children 1–4 years, and no clear reduction was observed in the unvaccinated under 5 s and the pre-vaccine 6-week age group. Our results suggest that although the current 3 + 0 schedule has had an impact on VT carriage in Malawi, different schedules including a booster dose may need to be assessed in order to obtain and maintain protection of the younger age groups until a more population-wide herd protection is achieved.
