# Supplementary material for: Pneumococcal carriage in households in Karonga District, Malawi, before and after introduction of 13-valent pneumococcal conjugate vaccination
Source: Vaccine. 2018 Nov 19;36(48):7369–76. doi: 10.1016/j.vaccine.2018.10.021 (PMC6238076; doi:10.1016/j.vaccine.2018.10.021)
Supplement: Supplementary data 1 [file mmc1.docx]

**Supplementary Material**

Heinsbroek et al., Pneumococcal carriage in households in Karonga District, Malawi, before and after introduction of 13-valent pneumococcal conjugate vaccination.

**Supplementary Figure 1: Overview of studies and analyses on nasopharyngeal samples of mothers, infants, and children 1-15 years in Karonga District, Malawi in the pre- and post-PCV13 periods.**

Longitudinal analyses for the pre-PCV13 period are available in Heinsbroek E, Tafatatha T, Chisambo C, et al. Pneumococcal Acquisition Among Infants Exposed to HIV in Rural Malawi: A Longitudinal Household Study. *Am J Epidemiol* 2016;183(1):70-8. NP = nasopharyngeal

**Survival analysis**

Pneumococcal acquisition in first 18 weeks (infants)

**2009-2011: pre-PCV13 introduction**

**2014: post-PCV13 introduction** *(samples collected April-August)*

**Pneumococcal prevalence:**

**- Infants:** 6 weeks & 18 weeks of age

**- Children 1-4 years^*^**

**- Children 5-15 years^*^**

- **HIV-negative mothers^*^**

**^*^***All longitudinal samples included for children 1-4 years and 5-15 years and mothers; within-person clustering adjusted for in mixed model analysis*

**Longitudinal component:**

NP sampling at **6, 8, 10, 12, 14, 16, 18 weeks** of infant age from:

- Infants

- Mothers

- Children 1-4 years in household

*Samples included if collected in months April-August*

**Longitudinal study:**

NP sampling at **6, 10, 14, 18, 22, 26, 34, 40, 46, 52 weeks** of infant age from:

- Infants

- Mothers

- Children 1-15 years in household

**Cross-sectional component:**

NP sampling at **6 weeks** of infant age from:

- Infants

- Mothers

- Children 1-15 years in household

Supplementary Figure 2. Kaplan-Meier plot for time to first pneumococcal carriage acquisition of any serotype in infants living with or without other children 1-4 years in Karonga District, Malawi, 2014 (post introduction of PCV-13).

Observations in the pre-vaccination period right-truncated at 18 weeks (maximum observation period post-vaccination period).

| Supplementary Table 1. Studies comparing pneumococcal carriage pre- and post-introduction of PCV in sub-Saharan African countries | | | | | | | |
| --- | --- | --- | --- | --- | --- | --- | --- |
| **Country** | **Vaccine introduction, schedule, catch-up** | **Comparison** | **Age group** | **VT carriage prevalence**  **pre-; post- vaccination period** | **Adjusted VT prevalence or risk ratio (95%CI)** | **NVT carriage prevalence pre-; post-vaccination period** | **Adjusted NVT prevalence or risk ratio (95%CI)** |
| Kenya ([1](#_ENREF_1), [2](#_ENREF_2)) | PCV10: 2011, 3+0  Catch-up: Kilifi Region: <5 years received up to two doses | 2009-2010 vs. 2011-2012 | <5 yrs  ≥5 yrs | 34%; 13% 8%; 4% | 0.36 (0.26-0.51)  0.34 (0.18-0.62) | 41%; 57% 24%; 27% | 1.37 (1.13-1.65)  1.13 (0.92-1.38) |
|  |  | 2009-2010 vs. 2012-2016 | <5 yrs  5-14 yrs  ≥15 yrs | 34%; 9%  15%; 6%  6%; 1% | 0.26 (0.19-0.35)  0.38 (0.22-0.64)  0.23 (0.12-0.44) | 41%; 70%  37%; 44%  18%; 23% | 1.71 (1.47-1.99)  1.25 (1.01-1.54)  1.30 (1.05-1.63) |
| South Africa ([3](#_ENREF_3)) | PCV7: 2009 &  PCV-13: 2011, 2+1  No catch-up campaign | 2009 vs. 2011 | <2 yrs  2-5 yrs  6-12 yrs  13-18 yrs  19-45 yrs  >45 yrs | 45%; 24%  36%; 29%  19%; 13%  6%; 2%  3%; 1%  2%; 1%  (estimates for PCV7) | 0.50 (0.42-0.59)  0.79 (0.64-0.99)  0.66 (0.48-0.92)  0.49 (0.17-1.39)  0.36 (0.18-0.74)  0.63 (0.16-2.49) | 39%; 50%  46%; 44%  43%; 38%  18%; 10%  8%; 4%  3%; 4% | 1.35 (1.17-1.56)  0.99 (0.84-1.18)  0.87 (0.73-1.04)  0.55 (0.34-0.89)  0.46 (0.31-0.68)  1.15 (0.48-2.72) |
| The Gambia ([4](#_ENREF_4)) | PCV7: 2009 &  PCV-13: 2011, 3+0  No formal catch-up, but most <2 years received one dose PCV7 | 2011 vs. 2012 | 6-11 mo  mothers | 33%; 18% PCV-13  9%; 5% PCV-7  7%; 8% PCV-13  3%; 3% PCV-7 | 0.52 (0.39-0.69)  0.52 (0.28-0.97)  1.14 (0.65-2.01)  0.85 (0.31-2.30) |  |  |

**References**

1. Hammitt L, Etyang AO, Morpeth SC, et al. Impact of 10-valent pneumococcal conjugate vaccine on invasive pneumococcal disease and nasopharyngeal carriage in Kenya. *bioRxiv* 2018;DOI 10.1101/369876.

2. Hammitt LL, Akech DO, Morpeth SC, et al. Population effect of 10-valent pneumococcal conjugate vaccine on nasopharyngeal carriage of Streptococcus pneumoniae and non-typeable Haemophilus influenzae in Kilifi, Kenya: findings from cross-sectional carriage studies. *Lancet Glob Health* 2014;2(7):e397-405.

3. Nzenze SA, Shiri T, Nunes MC, et al. Temporal changes in pneumococcal colonization in a rural African community with high HIV prevalence following routine infant pneumococcal immunization. *Pediatr Infect Dis J* 2013;32(11):1270-8.

4. Roca A, Bojang A, Bottomley C, et al. Effect on nasopharyngeal pneumococcal carriage of replacing PCV7 with PCV13 in the Expanded Programme of Immunization in The Gambia. *Vaccine* 2015;33(51):7144-51.
